# Additive Effects of Anxiety and Depression on Body Mass Index among Blacks: Role of Ethnicity and Gender

**Published:** 2014-04-01

**Authors:** Shervin Assari

**Affiliations:** 1 Department of Health Behavior and Health Education, School of Public Health, University of Michigan, Ann Arbor, MI, USA; 2 Center for Research on Ethnicity, Culture and Health, School of Public Health, University of Michigan, Ann Arbor, MI, USA

**Keywords:** Anxiety, Depression, Obesity, Body Mass Index, Race, Ethnic Groups, Gender

## Abstract

**Background:**

Most studies on mental health associates of obesity have focused on depression and less is known about the role of anxiety in obesity.

**Objectives:**

This study compared the additive effects of General Anxiety Disorder (GAD) and Major Depressive Disorder (MDD) on Body Mass Index (BMI) across sub-populations of Blacks based on the intersection of ethnicity and gender.

**Methods:**

Data came from the National Survey of American Life (NSAL), 2001 - 2003. The participants consisted of 3,570 African Americans and 1,621 Caribbean Blacks. Twelve-month MDD and GAD were determined using the World Mental Health Composite International Diagnostic Interview (CIDI). Levels of BMI were categorized based on being equal to or larger than 25, 30, 35, and 40 kg/m^2^. We fitted linear regression models specific for our groups, which were defined based on the intersection of ethnicity and gender. Additionally, age, education, marital status, employment, and region were controlled.

**Results:**

Among Caribbean Black men and African American women, lifetime GAD, but not MDD, was associated with high BMI. Among Caribbean Black women, lifetime MDD, but not GAD, was associated with high BMI.

**Conclusions:**

Intersection of ethnicity and gender may determine how anxiety and depression are associated with BMI among Blacks. Sub-populations of Blacks (e.g. based on ethnicity and gender) may have specific mental health determinants or consequences of obesity. Future research should investigate how and why the additive effects of anxiety and depression on obesity vary across ethnic and gender groups of Blacks.

## 1. Background

Anxiety and depressive disorders are among the most prevalent mental disorders in the United States and around the world (1-3). The U.S. National Comorbidity Survey Replication (NCS-R) in 2001 - 2003 reported lifetime prevalence of anxiety and depressive disorders to be 28.8% and 20.8%, respectively (3). Based on the same survey, 18.1% and 9.5% of U.S. adults have had anxiety and depressive disorders over the past twelve months (2).

Although anxiety may also be associated with obesity (4-10), most studies on the association between psychiatric disorders and obesity have exclusively studied depression (9, 11, 12). From the pool of studies reporting a link between anxiety and obesity, most have been conducted in the clinical setting, which results in enrollment of participants with higher levels of Body Mass Index (BMI), such as individuals with morbid or very severe obesity (7).

The studies that have focused on the association between psychiatric disorders (mostly depression) and obesity have provided mixed results (13). Consequently, researchers are now interested in exploring moderators that may be responsible for some of these inconsistencies. This body of research has suggested a wide range of candidate moderators, including but not limited to race (11, 12), ethnicity (11, 14), and gender (11, 15-19).

The literature is limited regarding the moderating effects of race and ethnicity on the psychiatric disorders that might be associated with obesity (20). As most surveys in the U.S. have predominantly sampled Whites (11), our knowledge is limited on the association between General Anxiety Disorder (GAD) / Major Depressive Disorder (MDD) and obesity among racial and ethnic minority groups. It is not known if race, ethnicity, and gender modify the links between psychiatric disorders and obesity or not (11, 12, 21). Gavin and colleagues analyzed the Comprehensive Psychiatric Epidemiology Surveys (CPES) data and showed Black-White differences in the association between 12-month MDD and obesity (11). Assari also showed that the direction and magnitude of the association between MDD and BMI among Blacks might depend on gender and ethnicity (21).

## 2. Objectives

The current study aimed to investigate the additive effects of lifetime GAD and MDD on BMI among Blacks based on the intersection of ethnicity and gender. In this study, we tested if four ethnic / gender groups differed regarding the association between lifetime GAD, lifetime MDD, and BMI level. The groups in this study composed of African American men, African American women, Caribbean Black men, and Caribbean Black women.

## 3. Patients and Methods

In this cross-sectional study, we used data from the National Survey of American Life (NSAL), 2001 - 2003. The NSAL is the largest mental health survey of Blacks ever conducted in the United States (22). The study is unique because it has provided a representative sample of Caribbean Blacks, the second major ethnic group of Blacks. The NSAL was conducted as a part of the Collaborative Psychiatric Epidemiology Surveys (CPES) funded by the National Institute of Mental Health (NIMH).

### 3.1. Ethics

The study protocol was approved by the Institute Review Board of the University of Michigan, Ann Arbor. All the participants provided written informed consent. The consents were received through mail from those whose data were collected via telephone interview. In addition, all the data were collected anonymously.

### 3.2. Participants

This study included 3,570 African Americans and 1,621 Caribbean Blacks who were sampled in the NSAL. African Americans and Caribbean Blacks were nationally representative of the Black individuals who were 18 years and older in the United States. Although African Americans were residents of either large cities or other urban and rural areas, Caribbean Blacks were only sampled from large cities. Detailed reports on the sampling have been provided elsewhere (23, 24). The overall response rate was 72.3%: 70.7% for the African Americans and 77.7% for the Caribbean Blacks.

### 3.3. Interview

Interviews lasted for 140 minutes on average. The data were collected using a Computer-Assisted Personal Interview (CAPI) for 86% of the individuals who participated in the study. The remaining interviews were either partially or entirely conducted via telephone. All the interviews were performed in English.

### 3.4. Measures

#### 3.4.1. Body Mass Index

The BMI level was calculated based on self-reported weights and heights. Weight and height were originally collected in pounds (1 pound = 0.453 kilograms) and feet (1 foot = 0.3048 meters) / inches (1 inch = 0.0254 meters), respectively. Then, BMI was categorized based the on the following cut-off points: equal to or larger than 25, 30, 35, and 40 kg/m^2^. Thus, BMI included under-weight, normal weight, obesity class I, obesity class II, and obesity class III.

BMI calculated based on self-reported weight and height is known to be closely correlated with BMI based on direct measures of height and weight (11). However, using self-reported weight and height may lead to some degrees of underestimation of BMI (25), because of a systematic tendency for humans to underestimate their weight and to overestimate their height (26).

#### 3.4.2. Lifetime MDD / GAD

Lifetime MDD and GAD were measured using the World Mental Health Composite International Diagnostic Interview (CIDI). The CIDI was developed for the World Mental Health project initiated in 2000 (27). The CIDI is used by trained lay interviewers to generate diagnoses of lifetime and recent DSM-IV-TR / ICD-10 disorders (28). Clinical reappraisal studies have documented high concordance of CIDI diagnoses with diagnoses made by psychiatrists (27, 29). Investigation of area under the receiver operating characteristic curve (AUC) has found excellent concordance between CIDI-SC and the Structured Clinical Interview for DSM-IV diagnoses of MDD and GAD. Additionally, the prevalence differences between CIDI-SC and Structured Clinical Interview for DSM-IV (SCID) are non-significant at the optimal CIDI-SC diagnostic thresholds. Thus, CIDI-SC operating characteristics are equivalent for MDE and GAD to those of the best alternative screening scales (30). CIDI is also known to provide valid findings for Blacks and their ethnic groups (31-33).

### 3.5. Control Variables

We measured socio-demographic characteristics such as age, employment status, education, and marital status. Country region was also controlled.

### 3.6. Statistical Analysis

We used Stata 13.0 for data analysis to account for the complex sampling design. The Taylor series approximation technique was used for calculating the standard errors. Standard errors reflect the recalculation of variance using the study's complex design. Moreover, linear regressions were used for multivariable analysis by considering lifetime MDD and GAD as predictors and BMI level as outcome. We conducted our analysis separately for each gender / ethnic group. Age, education, marital status, employment, and country region were entered to all models as control variables. P values less than 0.05 were considered as statistically significant. Adjusted Beta and 95% Confidence Interval (CI) were reported.

## 4. Results

In the present study, most participants in all ethnic groups were between 30 and 44 years of age. Almost half of the participants in each ethnic group were female. Most Caribbean Blacks were born outside the United States, while most African Americans were born inside the country. Although most African Americans lived in the South, the Northeast represented most of the Caribbean Blacks.

### 4.1. The Effect of Anxiety and Depression on BMI among Blacks

Among Blacks, anxiety but not depression was associated with BMI. African American ethnicity, female gender, education, and marital status were also associated with BMI among Blacks (Table 1). 

**Table 1.** The Association between Lifetime Major Depressive Disorder, General Anxiety Disorders and Body Mass Index (BMI) among Blacks

	B	SE ^a^	Sig	95% CI for B	
**Lifetime anxiety**	0.323	0.145	0.030	0.032	0.613
**Lifetime depression**	-0.052	0.079	0.517	-0.210	0.107
**African American**	0.231	0.053	< 0.001	0.125	0.336
**Female gender**	0.247	0.035	< 0.001	0.177	0.316
**Age**	0.003	0.002	0.055	0.000	0.007
**Employment** ^**b**^					
Unemployed	-0.121	0.085	0.161	-0.291	0.050
Not in labor force	-0.083	0.072	0.252	-0.228	0.061
**Education** ^**c**^					
12 years	-0.073	0.050	0.149	-0.174	0.027
13 - 15 years	-0.103	0.057	0.078	-0.218	0.012
16 years or more	-0.234	0.061	< 0.001	-0.356	-0.112
**Marital status** ^**d**^					
Divorced / Separated / Widowed	-0.152	0.054	0.007	-0.260	-0.043
Never married	-0.262	0.052	< 0.001	-0.367	-0.158
**Region** ^**e**^					
Midwest	0.158	0.090	0.084	-0.022	0.338
South	0.017	0.070	0.810	-0.124	0.157
West	-0.072	0.086	0.404	-0.243	0.100
**Intercept**	2.699	0.113	< 0.001	2.473	2.926

^a^Abbreviations: SE, Standard Error

^b^Reference group: Employed;

^c^Reference group: 11 years or less;

^d^Reference group: Married;

^e^Reference group: Northeast

### 4.2. The Effect of Anxiety and Depression on BMI among Caribbean Black Men

Among Caribbean Black men, anxiety was associated with BMI. However, no significant association was observed between BMI and education or marital status (Table 2). 

**Table 2.** The Association between Lifetime Major Depressive Disorder and General Anxiety Disorders and Body Mass Index (BMI) among Caribbean Black Men

	B	SE ^a^	Sig	95% CI for B	
**Lifetime anxiety**	0.661	0.283	0.029	0.076	1.246
**Lifetime depression**	­0.320	0.236	0.187	­0.808	0.167
**Age**	0.005	0.005	0.386	­0.006	0.016
**Employment** ^**b**^					
Unemployed	­0.113	0.118	0.348	­0.358	0.131
Not in labor force	­0.211	0.168	0.222	­0.558	0.136
**Education** ^**c**^					
12 years	0.100	0.223	0.657	­0.360	0.561
13 ­ 15 years	0.386	0.190	0.054	­0.006	0.778
16 years or more	0.327	0.162	0.056	­0.009	0.662
Marital status ^**d**^					
Divorced / Separated / Widowed	­0.271	0.191	0.169	­0.667	0.124
Never married	­0.193	0.225	0.399	­0.658	0.272
**Region** ^**e**^					
Midwest	0.277	0.671	0.684	­1.112	1.665
South	­0.171	0.118	0.162	­0.416	0.074
West	­0.028	0.310	0.929	­0.669	0.614
**Intercept**	2.632	0.356	< 0.001	1.897	3.368

^a^Abbreviations: SE, Standard Error

^b^Reference group: Employed;

^c^Reference group: 11 years or less;

^d^Reference group: Married;

^e^Reference group: Northeast

### 4.3. The Effect of Anxiety and Depression on BMI among Caribbean Black Women

Among Caribbean Black women, depression and marital status were associated with BMI. However, no significant association was found between education and BMI among this group (Table 3). 

**Table 3.** The Association between Lifetime Major Depressive Disorder and General Anxiety Disorders and Body Mass Index (BMI) among Caribbean Black Women

	B	SE ^a^	Sig	95% CI for B	
**Lifetime anxiety**	0.616	0.477	0.209	­0.370	1.602
**Lifetime depression**	0.648	0.178	0.001	0.279	1.017
**Age**	­0.007	0.006	0.293	­0.020	0.006
**Employment** ^**b**^					
Unemployed	­0.018	0.327	0.957	­0.694	0.658
Not in labor force	­0.015	0.122	0.901	­0.268	0.238
**Education** ^**c**^					
12 years	0.002	0.194	0.993	­0.400	0.404
13 ­ 15 years	­0.086	0.237	0.720	­0.575	0.404
16 years or more	0.028	0.220	0.899	­0.428	0.485
**Marital status ^d^**					
Divorced / Separated / Widowed	­0.308	0.134	0.031	­0.587	­0.030
Never married	­0.255	0.268	0.351	­0.810	0.300
**Region** ^**e**^					
Midwest	­0.197	0.134	0.156	­0.474	0.081
South	­0.180	0.181	0.331	­0.554	0.194
West	0.552	0.833	0.514	­1.170	2.275
**Intercept**	3.537	0.281	< 0.001	2.955	4.119

^a^Abbreviations: SE, Standard Error

^b^Reference group: Employed;

^c^Reference group: 11 years or less;

^d^Reference group: Married;

^e^Reference group: Northeast

### 4.4. The Effect of Anxiety and Depression on BMI among African American Men

Among African American men, neither anxiety nor depression was associated with BMI. Additionally, marital status and employment, but not education, were associated with BMI (Table 4). 

**Table 4.** The Association between Lifetime Major Depressive Disorder and General Anxiety Disorders and Body Mass Index (BMI) among African American Men

	B	SE ^a^	Sig	95% CI for B	
**Lifetime anxiety**	­0.151	0.216	0.487	­0.590	0.287
**Lifetime depression**	0.006	0.136	0.964	­0.271	0.283
**Age**	0.004	0.003	0.142	­0.001	0.009
**Employment** ^**b**^					
Unemployed	­0.080	0.142	0.574	­0.368	0.207
Not in labor force	­0.213	0.103	0.047	­0.423	­0.003
**Education** ^**c**^					
12 years	­0.068	0.080	0.396	­0.230	0.093
13 ­ 15 years	0.038	0.075	0.617	­0.114	0.189
16 years or more	0.123	0.113	0.283	­0.106	0.353
**Marital status** ^**d**^					
Divorced / Separated / Widowed	­0.265	0.088	0.005	­0.443	­0.086
Never married	­0.316	0.079	< 0.001	­0.477	­0.156
**Region** ^**e**^					
Midwest	0.200	0.181	0.278	­0.169	0.569
South	0.010	0.142	0.944	­0.278	0.298
West	­0.079	0.169	0.644	­0.423	0.265
**Intercept**	3.138	0.197	< 0.001	2.737	3.539

^a^Abbreviations: SE, Standard Error

^b^Reference group: Employed;

^c^Reference group: 11 years or less;

^d^Reference group: Married;

^e^Reference group: Northeast

### 4.5. The Effect of Anxiety and Depression on BMI among African American Women

Among African American women, anxiety but not depression was associated with BMI. Also, education, marital status, and region were all associated with BMI (Table 5). 

**Table 5.** The Association between Lifetime Major Depressive Disorder and General Anxiety Disorders and Body Mass Index (BMI) among African American Women

	B	SE ^a^	Sig	95% CI for B	
**Lifetime anxiety**	0.518	0.190	0.010	0.132	0.903
**Lifetime depression**	­0.083	0.097	0.396	­0.281	0.114
**Age**	0.003	0.003	0.296	­0.003	0.010
**Employment** ^**b**^					
Unemployed	­0.184	0.116	0.122	­0.420	0.052
Not in labor force	­0.031	0.083	0.707	­0.199	0.137
**Education** ^**c**^					
12 years	­0.088	0.060	0.153	­0.209	0.034
13 ­ 15 years	­0.240	0.096	0.017	­0.436	­0.045
16 years or more	­0.605	0.088	0.000	­0.784	­0.427
**Marital status** ^**d**^					
Divorced / Separated / Widowed	­0.067	0.078	0.393	­0.226	0.091
Never married	­0.224	0.063	0.001	­0.353	­0.095
**Region** ^**e**^					
Midwest	0.180	0.058	0.004	0.062	0.298
South	0.056	0.048	0.248	­0.041	0.154
West	­0.099	0.058	0.096	­0.216	0.018
**Intercept**	3.434	0.168	< 0.000	3.093	3.775

^a^Abbreviations: SE, Standard Error

^b^Reference group: Employed;

^c^Reference group: 11 years or less;

^d^Reference group: Married;

^e^Reference group: Northeast

## 5. Discussion

The current study aimed to explore how African American and Caribbean Black men and women are different regarding the additive effects of GAD and MDD on BMI level. Our findings suggested that the intersection of ethnicity and gender influences the residual effect of lifetime GAD on BMI, while lifetime MDD was controlled. Among Caribbean Black men and African American women, lifetime GAD was associated with high BMI. On the other hand, among Caribbean Black women, lifetime MDD was associated with high BMI.

Up to now, only very few available studies have investigated the effects of ethnicity and gender on mental health associates of obesity. A study by Gavin and colleagues suggested that among obese women, the prevalence of 12-month MDD was lower among Blacks compared to Whites. They also showed that the association between obesity and MDD was stronger among Whites compared to Blacks (11).

In 2014, Assari used the data from the NSAL and showed that the direction of the association between MDD and BMI among Blacks was reversed among men compared to women. Among Black men, there was a positive association between BMI and MDD, while the association was negative among Black women. The gradient effect of BMI level on MDD reached statistical significance only among a African American men. In addition, among women, the association between MDD and BMI ≥ 40 kg/m^2^ was stronger among Caribbean Blacks than African Americans (21). Gariepy and colleagues reported that obesity at baseline might reduce the likelihood of a subsequent major depression episode among men but not women (34).

A study in Australia showed considerable gender differences in the association between anxiety, depression, and BMI level. Among men, BMI was linked to depression symptoms and negative affect. However, the difference was only found between the under-weight and normal-weight groups. Under-weight men showed more depressive symptoms and negative affect. Interestingly, obese and over-weight men had fewer depressive symptoms and negative affect. Among women, BMI was associated with anxiety, depression, and negative affect. Similar to men, the under-weight women showed more depression and negative affect compared to the normal-weight women. Obese women, however, had better mental health, both for anxiety and negative affects. Similarly, over-weight women had lower anxiety, depression, and negative affect in comparison to the normal-weight women (5).

Sachs-Ericsson and colleagues reported a larger influence of BMI on depressive symptoms among Blacks than Whites (12). In contrast, analysis of the National Comorbidity Survey Replication Surve suggested a stronger association between obesity and MDD among Whites compared to non-Whites (25).

The conditions of MDD, GAD, and obesity are all pressing public health problems and result in enormous costs to society (35-37). Depression and anxiety are the leading causes of disability (38) and obesity is a major cause of morbidity and mortality (39). Major depressive disorder is one of the most prevalent psychiatric disorders in the United States, affecting over 16% of adults (40). All these emphasize the importance of our findings.

Results of the current study may help with reducing the burden of anxiety, depression, and obesity among African Americans and Caribbean Blacks. Lifetime prevalence of MDD has been reported to be 13% among Caribbean Blacks, which is 3% higher than that of African Americans (41). Compared to Whites, MDD tends to be more severe and disabling among Blacks. Depression is also less frequently diagnosed and treated among Blacks compared to Whites (41). We already know that race, ethnicity, and gender influence the pattern of mental healthcare need and the seeking for professional healthcare in the presence of a need. Caribbean Blacks less frequently use mental healthcare services compared to other ethnic groups (33, 42-44). With a consistent pattern across almost all US states, obesity is 50% more prevalent among Blacks compared to Whites (45).

Multiple hypotheses may explain why gender and ethnicity modify the association between mental health and obesity. Gender and ethnicity may shape how individuals change their food intake or physical activity in response to the presence of anxiety or depression. Change in any of these behaviors (e.g. eating or exercise) influences energy balance that may result in weight gain. Anxiety and depression may be differently presented among men and women in a certain ethnic group. Negative body image and stigma associated with obesity or psychiatric disorders may have specific consequences for health behaviors, social relations, or psychological distress among different ethnic groups (46).

Biological mechanisms behind the associations between socio-economic status, perceived stress, mental health, and obesity should also be investigated. Jackson proposed that alterations in hypothalamic-pituitary-adrenal axis activation due to socio-economic disadvantage, chronic stress, or excessive alcohol consumption may explain disparities in the rate of obesity in the United States. High levels of chronic stress may result in chronically raised circulating cortisol concentration that may be responsible for increased visceral fat depot among Blacks. Such adiposity may be a direct consequence of increase in adipogenesis or an indirect effect of central factors that increase appetite and food intake (47).

Our finding has implications for the elimination of ethnic and gender disparities in obesity in the United States. Knowledge about the pattern of the links among MDD, GAD, and obesity is essential for designing interventions that use a body weight maintenance approach for health promotion of Blacks (11).

Based on our findings, we invite clinicians to evaluate mental health of Blacks who are obese; however, the protocol of mental health screening of Blacks who are obese should be tailored to their ethnicity and gender. It has been suggested that effective management of obesity may require evaluation and treatment of comorbid psychiatric disorders (47). As suggested by previous researchers, prevention of obesity may benefit from evaluation and treatment of mental disorders (11, 48). We argue that prevention of obesity among Blacks may be more effective if tailored based on gender and ethnicity. Among Caribbean Black men and African American women, lifetime GAD may be more important among Blacks who are obese, while among Caribbean Black women, depression may need more attention.

The current study had a few limitations. Although previous studies have suggested that the association between BMI and psychiatric disorders may be non-linear (4), we only focused on the linear association between BMI and MDD / GAD. There is some evidence that the threshold effect may better explain these links compared to the linear (gradient) effects. Community and clinical samples may also differ in the pattern of associations between psychiatric disorders and obesity (49, 50). Moreover, the cross-sectional design of the study limited any inference about the cause and effect of our findings. Another limitation was that BMI was calculated based on self-reported weight and height. Finally, medical and psychiatric comorbidities were not included in the analysis.

Further research is needed to uncover the mechanisms by which the intersection of ethnicity and gender shapes how lifetime GAD and MDD are associated with obesity. Smoking, body image, perceived weight, feeling of guilt, intention for weight loss, eating habits, eating disorders, or physical activity may explain why anxiety and depression are differently associated with obesity. Many of these factors may potentially mediate the complex association between ethnicity, gender, depression, anxiety, and obesity. Psychosocial and lifestyle risk factors are common risk factors of obesity, MDD, and GAD, and their distributions vary based on race, ethnicity, and gender (47).

To conclude, the pattern of association between MDD, GAD, and BMI among Blacks in the U.S. may depend on the intersection of ethnicity and gender. Yet, further research is needed to determine the mechanisms behind such differences. Meanwhile, programs that aim to prevent or treat obesity among Blacks through mental health interventions should be tailored based on the individuals’ ethnicity and gender.
